# Reconstruction of an Early-Stage Scapholunate Advanced Collapse Wrist with the 3-Ligament Tenodesis Procedure: A Controversial Reappraisal

**DOI:** 10.1097/PRS.0000000000011290

**Published:** 2024-01-15

**Authors:** Kasper N. Dullemans, Mark J. W. van der Oest, Stefanie N. Hakkesteegt, Guus M. Vermeulen, J. Michiel Zuidam, Liron S. Duraku, Hand-Wrist Study Group, Reinier Feitz

**Affiliations:** Rotterdam and Amsterdam, the Netherlands; From the Departments of 1 Plastic, Reconstructive, and Hand Surgery; 2 Rehabilitation Medicine, Erasmus Medical Center; 3 Hand and Wrist Center, Xpert Clinic; 4 Department of Plastic, Reconstructive, and Hand Surgery, Amsterdam University Medical Center.

## Abstract

**Background:**

The authors hypothesized that the 3-ligament tenodesis (3-LT) procedure is still sufficient—even in scapholunate advanced collapse (SLAC) cases—to reduce pain and improve wrist function. The authors compared patient-reported outcomes of scapholunate interosseus ligament (SLIL) injury patients with SLAC, to SLIL injury patients treated with 3-LT, and then to patients who underwent proximal row carpectomy (PRC), as a control group.

**Methods:**

The authors included all patients with a traumatic SLIL injury and associated SLAC components treated with 3-LT and completed patient-rated wrist evaluation (PRWE) questionnaires preoperatively and at 12-month follow-up. First, the authors compared matched patients with SLIL injury and SLIL injury with SLAC, stage 1 to 3, who received 3-LT. Second, the authors compared patients who received 3-LT with patients who underwent PRC, while having SLAC stage 2 or 3.

**Results:**

The authors compared 51 patients with SLAC to 95 patients with SLIL injury who had a 3-LT procedure, and 10 3-LT patients were compared with 18 patients undergoing PRC, given SLAC stage 2 or 3. In both analyses, the PRWE scores had significantly improved in all groups; however, no significant differences in PRWE were found between 3-LT in SLIL injury and SLIL injury with SLAC (6.9 points; 95% CI, −14.92 to 1.22; *P* = 0.096) and between 3-LT and PRC, given SLAC stage 2 or 3, 15.1 points (not enough power).

**Conclusions:**

There is no difference in PRWE between matched SLIL injury patients with or without degenerative changes treated with a 3-LT. Therefore, the 3-LT procedure seems to be a viable treatment option for patients with early-stage SLAC wrist.

**CLINICAL QUESTION/LEVEL OF EVIDENCE:**

Therapeutic, III.

The scapholunate interosseous ligament (SLIL) is a key intercalating stabilizer of the wrist, ensuring normal motion of carpal bones.^1^ Traumatic injuries to the SLIL are mainly known for producing instability of the intercarpal joints, causing pain and dysfunction.^2^ Attenuation of the SL ligament will cause the scaphoid to abnormally deviate in a flexed position and the lunate pathologically in extension, which results in a pattern of dorsal intercalated segment instability.^3^ This condition leads to altered wrist kinematics and joint loading, causing damage to surrounding structures in the wrist, ultimately leading to degenerative osteoarthritis.^2,4,5^

The most common pattern of posttraumatic wrist arthritis involves the radioscaphoid and capitolunate joints, known as scapholunate advanced collapse (SLAC).^6^ Watson and Ballet introduced a classification system for SLAC wrist and identified different radiologic stages of degenerative osteoarthritis, which is predictable in its progress. The degenerative changes invariably progress from articulation between the distal scaphoid and radial styloid (stage 1) to the scaphoid fossa (stage 2) and finally involve the capitolunate joint (stage 3).^6,7^

The 3-ligament tenodesis (3-LT) procedure is a commonly used reconstructive technique to restore the normal alignment of carpal bones and possibly thereby prevent the occurrence of osteoarthritis; however, this is contraindicated when SLAC is present in the wrist. This is a core principle in hand surgery: when there is osteoarthritis, ligament reconstruction is not considered a treatment option.^8^ The accepted surgical treatments for a SLAC wrist are dependent on the stage and level of osteoarthritis. Multiple operative procedures are feasible in each SLAC stage, and local preferences exist on this matter, but generally, in the literature, it is recommended that a radial styloidectomy be performed in SLAC stage 1. In SLAC stage 2 and up, salvage procedures such as proximal row carpectomy (PRC), or midcarpal or total wrist fusion are usually performed.^3,9^ However, the PRC is the preferred and most commonly performed surgical treatment for SLIL injury with SLAC.^10^ In general, all these surgical options are salvage treatments and do not reconstruct the normal carpal anatomy.

However, it can be hypothesized that damage to the articular surface in a SLAC wrist may be limited and may not progress to the entire joint surface when carpal kinematics are restored. The 3-LT procedure relieves pressure on the osteoarthritic parts, reducing pain and improving wrist function. This would restore normal articulation, and possibly thereby prevent loss of congruence, and postpone salvage procedures. Therefore, this study aims to examine the patient-reported outcomes of SLIL injury patients with SLAC, stages 1 to 3, treated with 3-LT with 1-year follow-up. We compared them to the outcome of SLIL injury patients treated with 3-LT. Because PRC is a preferred procedure in SLIL injury with SLAC, we deemed it necessary to also compare 3-LT patients to patients who received a PRC, both when treated for SLAC wrist stage 2 or 3.

## PATIENTS AND METHODS

### Study Cohort

This observational cohort study looked at a subset of data from a database, which was also used for previously described studies^11,12^ at Xpert Clinics in the Netherlands. Xpert Clinics is a consortium comprising 22 outpatient clinics for hand surgery and therapy. Patients^13^ treated with 3-LT between December of 2011 and December of 2019 and who completed the patient-rated wrist evaluation (PRWE) questionnaire at intake, were included. The study was performed with the approval of the local medical ethics committee (Rotterdam, NL/sl/MEC-2018-1088). All patients had given prior informed consent to the anonymous use of their data.

Patients with a traumatic SL lesion were eligible for inclusion. Electronic patient files were assessed on medical history, and medical imaging (radiography, computed tomography, magnetic resonance imaging, and/or arthroscopy) reports were evaluated to identify the traumatic cause. A traumatic injury was defined as a complete rupture (Geissler 4) of the SL ligament or a partial rupture (Geissler 2 or 3) caused by a clear traumatic event.^14,15^ Patients were excluded if the 3-LT procedure was a reoperation, when the procedure was combined with another surgical treatment intervention, such as a styloidectomy or ulnar shortening osteotomy, or when another surgical procedure was performed within 8 weeks before or after the 3-LT operation. Patients with scaphoid nonunion collapse, perilunate wrist injuries, or Kienböck disease were also excluded from the analyses. Other exclusion criteria were midcarpal instability or laxity as a reason for the 3-LT procedure, and the presence of incomplete medical records. Wrist arthroscopies were considered a diagnostic procedure, and therefore, when arthroscopies were combined with or were performed within 8 weeks of the open 3-LT procedure, patients were not excluded. Fully arthroscope-assisted 3-LT procedures were excluded. As a control group, we included patients who received PRC for the indication SLAC stage 2 or 3.

### Surgical Technique

#### 3-Ligament Tenodesis

An experienced anesthetist applied regional block anesthesia. Our 3-LT technique is based on what was previously described by Garcia-Elias et al.^8^ First, the SL ligament is approached dorsally through the third and fourth extensor compartments of the wrist. Subsequently, a volar incision is made to expose the tubercle of the scaphoid and the flexor carpi radialis (FCR) tendon. A Kirschner wire is passed centrally through the scaphoid. A cannulated 2.7-mm drill bit is then used to drill a hole through the scaphoid in a longitudinal direction. Thereafter, a strip of the FCR tendon is passed through the scaphoid from volar to dorsal. Once the dorsal intercalated segment instability deformity is restored, a bone anchor (MITEK; Raynham, MA; JuggerKnot Soft Anchor; Zimmer Biomet, Warsaw, IN)^16^ is used to fixate the graft to the lunate. The JuggerKnot Soft Anchor is an all-suture device and lacks polyether ether ketone or other composite material, but uses a coreless sleeve and suture construct.^17^ The FCR strip is then passed through the radiotriquetral ligament, after which the FCR strip is sutured to itself. Kirschner wires were not used for the temporary fixation of carpal bones. A posterior interosseous nerve (PIN) neurectomy was part of the standard procedure. Postoperatively, patients were immobilized for 3 to 5 days, after which a personalized orthosis was given for up to 6 weeks during the day and up to 12 weeks at night, and they were offered a 3-month extensive hand rehabilitation program.

#### Proximal Row Carpectomy

The procedure is generally performed under regional block anesthesia. A dorsal approach by means of the fourth extensor compartment is used. A radially based flap/Berger flap is created to provide an adequate view of the proximal and distal carpal rows. Next, the scapholunate and lunotriquetral interosseous ligaments are severed, allowing the complete removal of the scaphoid, lunate, and triquetrum bones. Radial styloidectomy was not routinely performed; this was left to the discretion of the surgeons. A PIN resection was part of the standard procedure. The wrist capsule and skin were closed, and a volar plaster of Paris splint was applied. Postoperatively, patients were immobilized for 3 to 5 days, after which a personalized orthosis was given for up to 6 weeks during the day and up to 12 weeks at night, and they were offered a 3-month extensive hand rehabilitation program.

### Data Sources

The patient-reported outcomes were acquired from routine questionnaire administration in our practices using a process of electronic data collection previously described.^18^ Participants were asked to answer electronically based questionnaires regarding specific risk factors and patient-related outcome measures at baseline, 3 months, and 12 months postoperatively. Electronic patient records of 3-LT patients were searched for the presence of SLAC preoperatively. Two independent plastic surgeons evaluated the medical imaging and arthroscopic records of patients in whom SLAC was suspected or mentioned in their electronic records and subsequently classified the patient’s degenerative osteoarthritis into the corresponding stages. Complications and any additional surgical procedures for osteoarthritis in the follow-up of 3-LT were also recorded (eg, PRC, anterior and posterior interosseous neurectomy, or styloidectomy). If a PIN structure or neuroma was identified, re-resection was performed.

### Outcome Measures

The primary endpoint of this study was the change in PRWE total score from baseline to 12 months postoperatively. The PRWE is a questionnaire that consists of 15 questions related to patients’ pain and the functionality of the wrist. Participants score these questions on a scale ranging from 0 (no pain or dysfunction) to 10 (severe pain and dysfunction).^19^ Pain and function scores together constitute the total score. High PRWE scores indicate more pain or dysfunction. Change in PRWE was calculated by subtracting the total score at intake from the total score at 12 months postoperatively.

Secondary endpoints were complications and conversion rate to additional surgical treatment of osteoarthritis in the wrist in the follow-up of 3-LT. Complications were scored following the International Consortium for Health Outcome Measurement Complications in Hand and Wrist Conditions classification, which is modified from the Clavien-Dindo classification for general surgery.^20^ This tool classifies complications within 12 months after surgery into different grades based on the treatment it requires. When a complication is not sufficiently relieved with minimally invasive treatment and more invasive treatment was given, only the complication with the highest grade is reported. For grade 3 complications (additional surgery), a longer follow-up was established. Patients were followed up for up to 9 years, with a median of 4.7 years (IQR, 2.7 to 6.7 years).

### Power

We performed an a priori power calculation for our primary analysis comparing SLIL injury patients and SLIL injury patients with SLAC components, both treated with a 3-LT, at one single time point. When using a 2:1 allocation rate and an α of 0.05 and a power of 0.8, we would have been able to detect an effect size of 0.5 or greater between the groups of 48 and 96. This would only have been an effect size of 0.95 or greater for the comparison with the PRC group. Any analysis with fewer participants would be underpowered and therefore not formally tested.

### Bias

Confounding was likely to be present, which risks the genuine association between the presence of SLAC and PRWE outcomes after 3-LT to be distorted. Propensity score matching (PSM) was applied to have comparable groups. The baseline characteristics used to match participants were age, sex, duration of symptoms, dominant hand, profession (heavy/moderate/light/no), pain, and function score at intake. In the second analysis, participants were also matched on the SLAC stage. All variables were weighted equally in the calculation. We used a 1:2 matching ratio and the nearest neighbor technique from the MatchIt package in R.^21^ If no matches were found, the associated data were omitted from the analyses.

### Statistical Analyses

In this study, we carried out two analyses. First, between SLIL injury patients and SLIL injury patients with SLAC stage 1 to 3, both treated with a 3-LT. Second, between patients who received a 3-LT and patients who underwent a PRC, while having SLAC stage 2 or 3. We performed separate PSM for each analysis (Tables 1 and 2).

**Table 1.** Baseline Characteristics Used for Matching Patients with SLIL Injury and SLIL Injury with SLAC Treated with 3-LT

	Before Matching (*n* = 311)			After Matching (*n* = 146)		
	3-LT (%)	3-LT plus SLAC (%)	*P*	3-LT (%)	3-LT plus SLAC (%)	*P*
No. of patients	258	53		95	51	
Mean age ± SD, hr	47.46 ± 12.05	52.06 ± 10.12	0.010	52.25 ± 10.86	51.33 ± 9.60	0.613
Sex			0.141			0.182
Female	114 (44.2)	17 (32.1)		42 (44.2)	16 (31.4)	
Male	144 (55.8)	36 (67.9)		53 (55.8)	35 (68.6)	
Mean duration of symptoms ± SD, mo	21.92 ± 43.0	27.98 ± 40.4	0.347	25.26 ± 45.39	25.31 ± 38.53	0.995
Mean pain score at intake ± SD	30.57 ± 10.5	28.25 ± 11.1	0.148	28.95 ± 10.6	28.53 ± 10.9	0.823
Mean function score at intake ± SD	27.41 ± 11.7	22.66 ± 13.5	0.010	23.26 ± 11.1	22.73 ± 13.3	0.796
SLAC						
0	258 (100.0)	0 (0.0)		95 (100.0)	0 (0.0)	
1	0 (0.0)	39 (73.5)		0 (0.0)	39 (76.5)	
2	0 (0.0)	11 (20.8)		0 (0.0)	10 (19.6)	
3	0 (0.0)	3 (5.7)		0 (0.0)	2 (3.9)	
Dominant hand			1.000			0.741
No	114 (44.2)	23 (43.4)		48 (50.5)	28 (54.9)	
Yes	144 (55.8)	30 (56.6)		47 (49.5)	23 (45.1)	
Profession			0.682			0.772
No	49 (19.0)	14 (26.4)		29 (30.5)	12 (23.5)	
Light	86 (33.3)	16 (30.2)		29 (30.5)	16 (31.4)	
Moderate	65 (25.2)	12 (22.6)		17 (17.9)	12 (23.5)	
Heavy	58 (22.5)	11 (20.8)		20 (21.1)	11 (21.6)	

**Table 2.** Baseline Characteristics Used for Matching Patients with SLAC Stage 2 or 3 Treated with 3-LT or PRC

	Before Matching (*n* = 141)			After Matching (*n* = 28)		
	PRC plus SLAC 2 or 3 (%)	3-LT plus SLAC 2 or 3 (%)	*P*	PRC plus SLAC 2 or 3 (%)	3-LT plus SLAC 2 or 3 (%)	*P*
No. of patients	127	14		18	10	
Mean age ± SD, yr	60.94 ± 9.61	50.21 ± 11.14	<0.001	57.11 ± 7.52	55.40 ± 5.38	0.532
Sex			0.589			1.000
Female	38 ± 29.9	6 ± 42.9		8 ± 44.4	5 ± 50.0	
Male	88 ± 69.3	8 ± 57.1		10 ± 55.6	5 ± 50.0	
Unknown	1 ± 0.8	0 ± 0.0		0 ± 0.0	0 ± 0.0	
Mean duration of symptoms ± SD, mo	41.24 ± 55.57	49.50 ± 69.35	0.608	24.89 ± 19.96	35.30 ± 45.35	0.405
Mean pain score at intake ± SD	32.12 ± 9.89	28.14 ± 9.69	0.155	25.72 ± 11.56	29.90 ± 9.45	0.339
Mean function score at intake ± SD	27.10 ± 11.49	21.29 ± 13.47	0.079	20.22 ± 11.16	21.90 ± 15.26	0.741
SLAC			0.239			1.000
2	117 (92.1)	11 (78.6)		15 (83.3)	8 (80.0)	
3	10 (7.9)	3 (21.4)		3 (16.7)	2 (20.0)	
Dominant hand			1.000			0.362
No	52 (40.9)	6 (42.9)		3 (16.7)	4 (40.0)	
Yes	75 (59.1)	8 (57.1)		15 (83.3)	6 (60.0)	
Profession			0.263			0.880
No	59 (46.5)	4 (28.6)		6 (33.3)	4 (40.0)	
Light	23 (18.1)	5 (35.7)		6 (33.3)	2 (20.0)	
Moderate	25 (19.7)	4 (28.6)		5 (27.8	3 (30.0)	
Heavy	20 (15.7)	1 (7.1)		1 (5.6)	1 (10.0)	

Paired *t* tests were performed to determine the statistical significance of the change in PRWE within a group between 2 time points (12 months postoperatively and preoperatively). Unpaired *t* tests were performed to calculate the significance of the change in PRWE between the groups in the analyses.

The Kaplan-Meyer method was used to assess the conversion rate to additional surgical treatment of osteoarthritis according to SLAC classification (stage 0 to 3) after 3-LT was performed. Patients were censored from the analysis when a competing event took place after 3-LT, which could itself have been the provoking factor for additional treatment of dealing with complaints of osteoarthritis. In addition, patients whose follow-up ended early because of unforeseen reasons, such as travel abroad, were censored by the time of last available data.

The statistical level of significance was defined as a value of *P* < 0.05. All analyses were performed using R version 3.6.0.

## RESULTS

### 3-LT in SLIL Injury versus 3-LT in SLIL Injury with SLAC

**Fig. 1.**
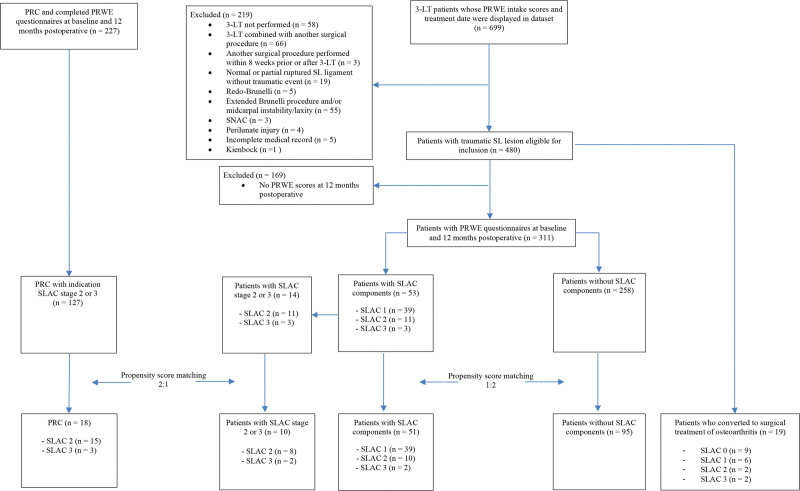
Flowchart. *SLAC*, scapholunate advanced collapse.

**Fig. 2.**
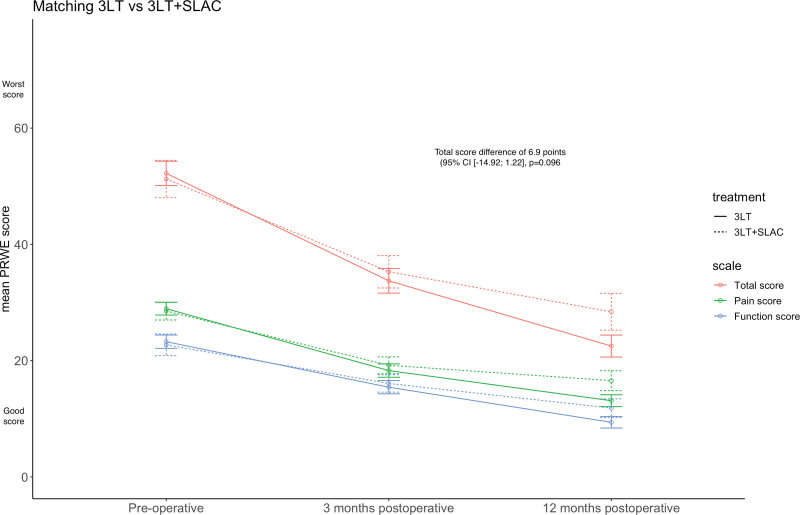
PRWE scores.

In total, 311 of 480 patients who were treated with 3-LT during the study period were eligible for inclusion. Of these 311 participants, 258 patients did not show any signs of SLAC, and 53 patients were diagnosed with SLAC (Fig. 1). Table 1 shows the differences in baseline characteristics in the prematch and postmatch comparison. After matching, the PRWE scores at 1-year follow-up had significantly improved in both groups, SLIL injury and SLIL injury with SLAC, respectively, 30 points (*P* < 0.05) versus 23 points (*P* < 0.05). Furthermore, no significant difference in PRWE between the matched groups was found after 12-month follow-up (6.9 points; 95% CI, –14.92 to 1.22; *P* = 0.096) (Fig. 2).

### 3-LT with SLAC Stage 2 or 3 versus PRC with SLAC Stage 2 or 3

**Fig. 3.**
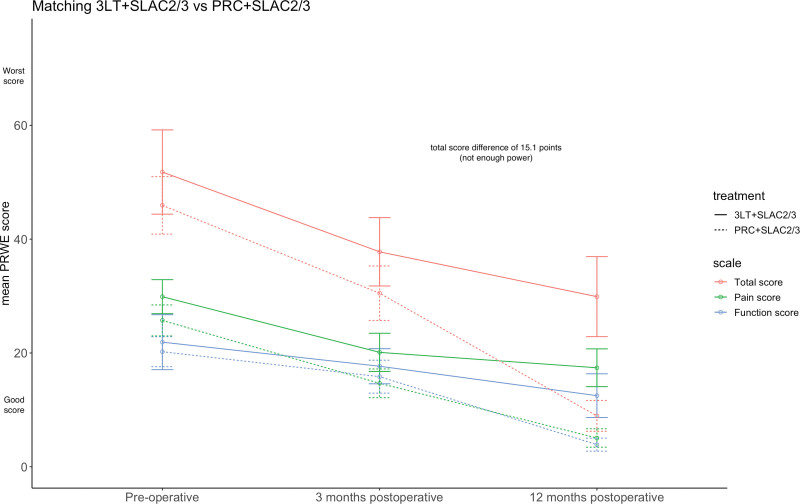
PRWE scores.

In the second analysis, 10 of 14 patients who were treated with a 3-LT were compared with 18 of 127 patients who underwent a PRC, while having SLAC stage 2 or 3 (Fig. 1). Table 2 presents the differences in characteristics between both groups before and after the matching procedure. After PSM at 12-month follow-up, the PRWE scores had significantly improved in both groups, 3-LT and PRC in SLAC 2 or 3, respectively, 21.9 points (*P* < 0.05) and 37.0 points (*P* < 0.05) (Fig. 3). We did not formally test the difference between these groups, because these groups’ sizes did not meet the required power.

### Complications

Table 3 shows the complications of all patients with a traumatic SLIL injury treated with a 3-LT for all stages of SLAC. According to the International Consortium for Health Outcome Measurement Complications in Hand and Wrist Conditions system, 15% of patients had grade 1 complications, 12% had grade 2 complications, and 12% had grade 3 complications (Table 3). The percentage of complications varies between groups, but grade 1 and 2 complications do not seem to be more prevalent in a specific group.

**Table 3.** Complications by International Consortium for Health Outcome Measurement Complications in Hand and Wrist Conditions Grade

SLAC	Grade 1			Grade 2	Grade 3
	Brace (%)	Infection (%)	Therapy (%)	Injection (%)	Surgery (%)
0	3	2	11	13	9
1	7	0	20	7	11
2	0	0	8	15	18
3	0	0	20	0	50

### Conversion Rate to Additional Surgery (Grade 3 Complications)

In total, 19 of 480 patients converted to an additional surgical osteoarthritis treatment after the 3-LT procedure during the median follow-up of 4.5 years (IQR, 2.7 to 6.7 years) (Fig. 1). Of these 19 patients, 13 participants received a PRC in their follow-up of 3-LT. In 4 patients, an additional neurectomy of the posterior and anterior interosseous nerve was conducted. In addition, 1 patient received a radial styloidectomy to address osteoarthritis. Lastly, 1 patient received a scaphoid excision after being treated with a 3-LT.

SLAC components in the wrist were identified preoperatively in 10 of these 19 patients (Fig. 1). At maximum follow-up of 9 years, 14.7% of all patients identified with SLAC (*n* = 80) converted to an additional surgical osteoarthritis treatment after the 3-LT procedure; however, patients with higher SLAC stages converted more frequently. The conversion rate was 2.3% in SLAC 0 (*n* = 400), 11.2% in SLAC 1 (*n* = 62), 18.2% in SLAC 2 (*n* = 13), and 50.0% in SLAC 3 (*n* = 5). (**See Figure, Supplemental Digital Content 1**, which shows reversed Kaplan-Meier plot, http://links.lww.com/PRS/H101.)

## DISCUSSION

This study aimed to investigate whether the 3-LT procedure is a viable treatment for traumatic SLIL injury patients with SLAC-stage wrists. Therefore, we examined the change in PRWE from intake to 12 months postoperatively and compared it with SLIL injury patients treated with a 3-LT and patients who received a PRC, while using PSM. All groups had significantly improved PRWE scores at 12 months postoperatively. No significant differences were found in PRWE total score in the first year postoperatively between the matched 3-LT patients with SLIL injury and SLIL injury with SLAC wrists. Our findings support the hypothesis that the 3-LT can be valuable in SLAC patients, particularly those with stages 1 and 2. However, because of the limitations of observational research, we are cautious to draw any definitive conclusions that can only be obtained in a randomized controlled trial.

The improvement in our cohort is comparable to previously reported patient-reported outcome measure after 3-LT. A previous study showed a significant improvement in PRWE scores between 0 and 12 months postoperatively with continuous improvement in patients who underwent 3-LT for SLIL injury. A PRWE total score improvement of 31 points was found,^22^ which is similar to the 30-point improvement of the matched 3-LT patients with SLIL injury in our study. This indicates that the matched cohort is a good representation of the patients treated with 3-LT because of SLIL injury. Studies by Athlani et al.^23^ and Pauchard et al.^24^ found an improvement in the PRWE of approximately 22 points and the Quick Disabilities of the Arm, Shoulder and Hand questionnaire of approximately 18 points in their follow-up of patients treated with 3-LT, which seems to be slightly worse than the patients identified with SLAC stage 1 to 3 in our present study.

**Fig. 4.**
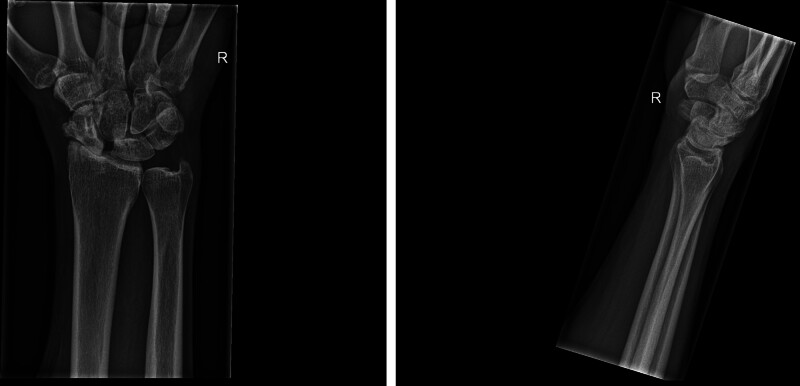
Posteroanterior and lateral wrist radiographs 6 years after index surgery, 3-LT for SLAC stage 3. There is a radiologic progression of osteoarthritis visible; clinically, the patient maintained all his activities.

Currently, the PRC or midcarpal fusion is the standard treatment for SLAC stage 2 or 3 wrists.^3,9^ The choice between a PRC and a 4-corner arthrodesis depends on the preference of the surgeon, as the outcomes of both procedures do not differ significantly; nevertheless, there may be a slight preference for a PRC.^10^ A PRC is considered a reliable procedure with significant improvements in the PRWE and the Disabilities of the Arm, Shoulder and Hand questionnaire, and preserved range of motion for the treatment of wrist arthritis and carpal trauma.^25,26^ However, long-term outcomes in the follow-up of PRC can also be disappointing, with the possible progression of osteoarthritis in the lunate fossa because of malalignment of the neo–capitate-radial joint resulting in significant pain and reduction of hand function.^27^ A 3-LT procedure may not completely halt the ongoing osteoarthritis process or prevent conversion to salvage procedures.^28^ However, we do believe that by restoring normal carpal kinematics, the 3-LT procedure will delay the course of normal progression of osteoarthritis in SLAC wrists,^29^ as shown in the postoperative radiographs (Fig. 4), thereby saving salvage procedures, such as a PRC, for later, because these are still feasible after a 3-LT is performed,^5^ and postponing its unfavorable long-term outcomes. As part of the shared decision-making process, the possibility of additional surgery should be pointed out to the patient.

In our study, the added value of 3-LT in SLAC patients seems to be limited to lower SLAC stages. In SLAC 1 and 2, the percentage of conversions to additional surgical procedures in the follow-up of 3-LT remains limited. During a median follow-up of 4.7 years (IQR, 2.7 to 6.7 years), conversion was 11.2% and 18.2%, respectively. However, patients classified with SLAC stage 3 converted more frequently, up to 50%. It is questionable whether performing a 3-LT in SLAC stage 3 is problem-solving; cost-effective; and therefore, of added value. Because of the high conversion rate, we will be more cautious to offer 3-LT in SLAC stage 3. In these particular patients, 3-LT could still be useful to postpone additional osteoarthritis surgery, but with this study, these patients can be properly counseled.

Our study has several strengths. First, this study uses longitudinal clinical data. The information gathered represents the results of the 3-LT treatment as part of daily clinical care. During these routine outcome measures, we noticed that surgeons would sometimes choose to perform a 3-LT in a selected group of SLAC wrist patients. Second, with the current study, we contribute to a subject that has been scarcely reported in the literature. Performing a 3-LT (without radial styloidectomy) while SLAC is present is controversial and might therefore not have been described in the current literature.

Our study has several limitations. First, SLIL injury patients with SLAC treated with 3-LT had less pain and better hand function compared with patients with SLIL injury treated with 3-LT and patients who received a PRC (Table 1). This was unexpected because we hypothesized that patients with associated damage to the articular surface of the wrist would have more preoperative complaints. This indicates that surgeons most likely performed a 3-LT instead of a PRC in a selected group of SLIL injury patients who had less pain and fewer functional problems. Understandably, surgeons are hesitant to perform a PRC when patients do not have pain and/or good hand function. However, this might lead to selection bias in our cohort. By using PSM, we adjust for these preoperative differences, try to mitigate this bias, and compare groups that are equal at baseline. A future randomized controlled trial is the only way to prevent this bias and could in turn provide a definitive answer to our research question. Second, only a limited number of people in the database were identified with a high classification of SLAC stage 2 or 3, which implies that it is not a common procedure for reconstruction, like 3-LT, in the case of advanced osteoarthritis in the wrist. As a result, the statistical power of the analyses is too low to perform an analysis at the final time point. Nevertheless, with a difference of 15.1 points in PRWE between the 3-LT and PRC group at 1 year postoperatively, the trend indicates that PRC gives a faster and better improvement of symptoms than 3-LT in SLIL injury patients with higher SLAC stages.

## CONCLUSIONS

Change in PRWE after 3-LT does not differ significantly between matched SLIL injury patients and SLIL injury patients with SLAC after 1-year follow-up. Furthermore, the complication and conversion rates of SLAC 1 and 2 in the follow-up of 3-LT remain limited. Moreover, salvage treatments such as PRC are always possible after a 3-LT procedure fails. Therefore, 3-LT can be considered a reconstruction treatment for early-stage SLAC wrists.

## DISCLOSURE

The authors have no financial conflicts of interest to disclose.

## Supplementary Material



## APPENDIX

The collaborators of the Hand-Wrist Study Group are as follows: R. A. M. Blomme, D. J. J. C. van der Avoort, A. Kroeze, J. Smit, J. Debeij, G. M. van Couwelaar, Guus M. Vermeulen, J. P. de Schipper, J. F. M. Temming, J. H. van Uchelen, H. L. de Boer, K. Harmsen, O. T. Zöphel, T. M. Moojen, X. Smit, R. van Huis, P. Y. Pennehouat, K. Schoneveld, Y. E. van Kooij, J. J. Veltkamp, A. Fink, W. A. de Ridder, H. P. Slijper, J. Tsehaie, R. Poelstra, M. C. Janssen, J. S. Teunissen, Jak Dekker, M. ter Stege, R. M. Wouters, J. M. Zuidam, L. Hoogendam, and W. R. Bijlsma.

## References

[1] Lee DJ Elfar JC. Carpal ligament injuries, pathomechanics, and classification. Hand Clin. 2015;31:389–398. 26205700 10.1016/j.hcl.2015.04.011 PMC4514919

[2] Weiss KE Rodner CM. Osteoarthritis of the wrist. J Hand Surg Am. 2007;32:725–746. 17482013 10.1016/j.jhsa.2007.02.003

[3] Konopka G Chim H. Optimal management of scapholunate ligament injuries. Orthop Res Rev. 2018;10:41–54. 30774459 10.2147/ORR.S129620 PMC6209348

[4] Murphy BD Nagarajan M Novak CB Roy M McCabe SJ. The epidemiology of scapholunate advanced collapse. Hand (N Y) 2020;15:23–26. 30003815 10.1177/1558944718788672 PMC6966289

[5] Kuo CE Wolfe SW. Scapholunate instability: current concepts in diagnosis and management. J Hand Surg Am. 2008;33:998–1013. 18656780 10.1016/j.jhsa.2008.04.027

[6] Watson HK Ballet FL. The SLAC wrist: scapholunate advanced collapse pattern of degenerative arthritis. J Hand Surg Am. 1984;9:358–365. 6725894 10.1016/s0363-5023(84)80223-3

[7] McLean A Taylor F. Classifications in brief: Watson and Ballet classification of scapholunate advanced collapse wrist arthritis. Clin Orthop Relat Res. 2019;477:663–666. 30179931 10.1097/CORR.0000000000000451 PMC6382201

[8] Garcia-Elias M Lluch AL Stanley JK. Three-ligament tenodesis for the treatment of scapholunate dissociation: indications and surgical technique. J Hand Surg Am. 2006;31:125–134. 16443117 10.1016/j.jhsa.2005.10.011

[9] Laulan J Marteau E Bacle G. Wrist osteoarthritis. Orthop Traumatol Surg Res. 2015;101(Suppl):S1–S9. 25596986 10.1016/j.otsr.2014.06.025

[10] Ahmadi AR Duraku LS van der Oest MJW Hundepool CA Selles RW Zuidam JM. The never-ending battle between proximal row carpectomy and four corner arthrodesis: a systematic review and meta-analysis for the final verdict. J Plast Reconstr Aesthet Surg. 2022;75:711–721. 34802951 10.1016/j.bjps.2021.09.076

[11] Hakkesteegt SN van der Oest MJW Dullemans KN; Hand-Wrist Study Group. Comparing patient-reported outcomes on three-ligament tenodesis between partial and complete scapholunate ligament injuries: a cohort study. J Hand Surg Am. 2024;49:712.e1–712.e9. 10.1016/j.jhsa.2022.09.012 36456426

[12] Teunissen JS Duraku LS Feitz R. Routinely-collected outcomes of proximal row carpectomy. J Hand Surg Am. 2024;49:795.e1–795.e9. 10.1016/j.jhsa.2022.09.004 36372595

[13] Feitz R van Kooij YE Ter Stege MHP; Hand-Wrist Study Group. Closing the loop: a 10-year experience with routine outcome measurements to improve treatment in hand surgery. EFORT Open Rev. 2021;6:439–450. 34267934 10.1302/2058-5241.6.210012 PMC8246110

[14] Sousa M Aido R Freitas D Trigueiros M Lemos R Silva C. Scapholunate ligament reconstruction using a flexor carpi radialis tendon graft. J Hand Surg Am. 2014;39:1512–1516. 24932851 10.1016/j.jhsa.2014.04.031

[15] Geissler WB. Arthroscopic management of scapholunate instability. J Wrist Surg. 2013;2:129–135. 24436805 10.1055/s-0033-1343354 PMC3699262

[16] Ruch DS Yang CC Smith BP. Results of acute arthroscopically repaired triangular fibrocartilage complex injuries associated with intra-articular distal radius fractures. Arthroscopy 2003;19:511–516. 12724681 10.1053/jars.2003.50154

[17] Agrawal V Pietrzak WS. Triple labrum tears repaired with the JuggerKnot soft anchor: technique and results. Int J Shoulder Surg. 2015;9:81–89. 26288537 10.4103/0973-6042.161440 PMC4528288

[18] Selles RW Wouters RM Poelstra R; Hand-Wrist Study Group. Routine health outcome measurement: development, design, and implementation of the hand and wrist cohort. Plast Reconstr Surg. 2020;146:343–354. 32740587 10.1097/PRS.0000000000007008

[19] MacDermid JC Turgeon T Richards RS Beadle M Roth JH. Patient rating of wrist pain and disability: a reliable and valid measurement tool. J Orthop Trauma 1998;12:577–586. 9840793 10.1097/00005131-199811000-00009

[20] Clavien PA Barkun J de Oliveira ML. The Clavien-Dindo classification of surgical complications: five-year experience. Ann Surg. 2009;250:187–196. 19638912 10.1097/SLA.0b013e3181b13ca2

[21] Ho D Imai K King G Stuart EA. MatchIt: nonparametric preprocessing for parametric causal inference. J Stat Softw. 2011;42:1–28.

[22] Blackburn J van der Oest MJW Poelstra R Selles RW Chen NC Feitz R; Hand-Wrist Study Group. Three-ligament tenodesis for chronic scapholunate injuries: short-term outcomes in 203 patients. J Hand Surg Eur Vol. 2020;45:383–388. 31711344 10.1177/1753193419885063

[23] Athlani L Pauchard N Dap F Dautel G. Treatment of chronic scapholunate instability: results with three-ligament tenodesis vs. scapholunate and intercarpal ligamentoplasty. Hand Surg Rehabil. 2019;38:157–164. 30904495 10.1016/j.hansur.2019.03.002

[24] Pauchard N Dederichs A Segret J Barbary S Dap F Dautel G. The role of three-ligament tenodesis in the treatment of chronic scapholunate instability. J Hand Surg Eur Vol. 2013;38:758–766. 23400768 10.1177/1753193413475753

[25] Chim H Moran SL. Long-term outcomes of proximal row carpectomy: a systematic review of the literature. J Wrist Surg. 2012;1:141–148. 24179718 10.1055/s-0032-1329547 PMC3658690

[26] Mulford JS Ceulemans LJ Nam D Axelrod TS. Proximal row carpectomy vs four corner fusion for scapholunate (Slac) or scaphoid nonunion advanced collapse (Snac) wrists: a systematic review of outcomes. J Hand Surg Eur Vol. 2009;34:256–263. 19369301 10.1177/1753193408100954

[27] Ali MH Rizzo M Shin AY Moran SL. Long-term outcomes of proximal row carpectomy: a minimum of 15-year follow-up. Hand (N Y) 2012;7:72–78. 23449142 10.1007/s11552-011-9368-y PMC3280369

[28] Goeminne S Borgers A van Beek N De Smet L Degreef I. Long-term follow-up of the three-ligament tenodesis for scapholunate ligament lesions: 9-year results. Hand Surg Rehabil. 2021;40:448–452. 33878482 10.1016/j.hansur.2021.03.020

[29] Andersson JK. Treatment of scapholunate ligament injury: current concepts. EFORT Open Rev. 2017;2:382–393. 29071123 10.1302/2058-5241.2.170016 PMC5644424

