# Supplementary material for: Reconstruction of an Early-Stage Scapholunate Advanced Collapse Wrist with the 3-Ligament Tenodesis Procedure: A Controversial Reappraisal
Source: Plast Reconstr Surg. 2024 Jan 15;154(5):1015–24. doi: 10.1097/PRS.0000000000011290 (PMC11512609; doi:10.1097/PRS.0000000000011290)
Supplement: Supplementary file 1 [file prs-154-01015-s001.pdf]

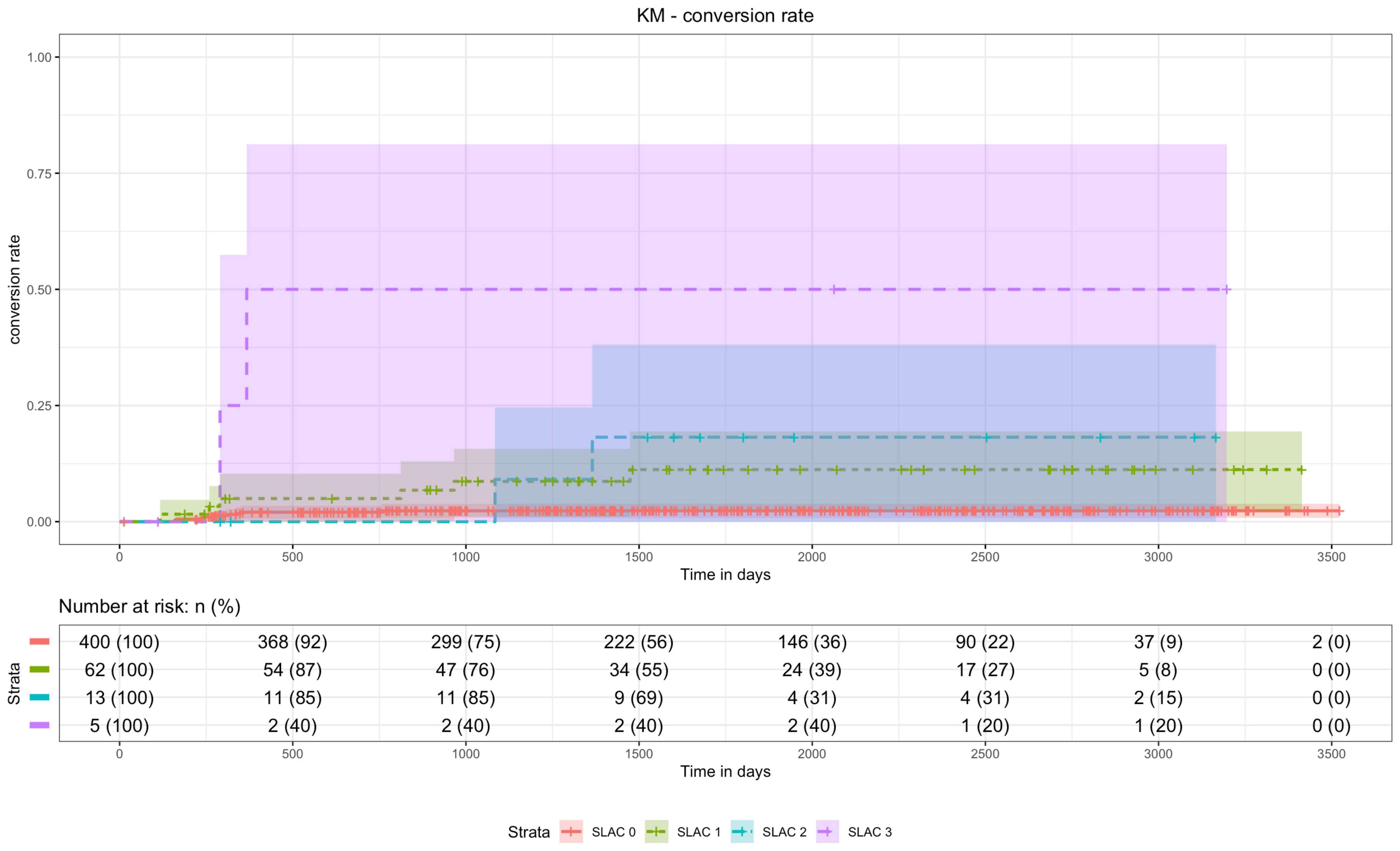
KM - conversion rate
conversion rate
1.00
0.75
0.50
0.25
0.00
0
500
1000
1500
2000
2500
3000
3500
Time in days
Number at risk: n (%)
Strata
400 (100) 368 (92) 299 (75) 222 (56) 146 (36) 90 (22) 37 (9) 2 (0)
62 (100) 54 (87) 47 (76) 34 (55) 24 (39) 17 (27) 5 (8) 0 (0)
13 (100) 11 (85) 11 (85) 9 (69) 4 (31) 4 (31) 2 (15) 0 (0)
5 (100) 2 (40) 2 (40) 2 (40) 2 (40) 1 (20) 1 (20) 0 (0)
0
500
1000
1500
2000
2500
3000
3500
Time in days
Strata
SLAC 0
SLAC 1
SLAC 2
SLAC 3
